# Role of long non-coding RNA ELFN1-AS1 in carcinogenesis

**DOI:** 10.1007/s12672-024-00929-x

**Published:** 2024-03-13

**Authors:** Zanyar HajiEsmailpoor, Alireza Fayazi, Mohammadhossein Teymouri, Peyman Tabnak

**Affiliations:** 1 Faculty of Medicine, Tabriz University of Medical Sciences, Tabriz, Iran; 2 Department of Metal Engineering, Cellular and Molecular Biology, Islamic Azad University Najafabad Branch, Isfahan, Iran; 3 Department of Biology and Biotechnology, University of Pavia, Pavia, Italy

**Keywords:** lncRNA, microRNA, Non-coding RNA, Cancer, Prognosis

## Abstract

As one of the leading causes of death worldwide, cancer significantly burdens patients and the healthcare system. The role of long non-protein coding RNAs (lncRNAs) in carcinogenesis has been extensively studied. The lncRNA ELFN1-AS1 was discovered recently, and subsequent studies have revealed its aberrantly high expression in various cancer tissues. In vitro and in vivo experiments have consistently demonstrated the close association between increased ELFN1-AS1 expression and malignant tumor characteristics, particularly in gastrointestinal malignancies. Functional assays have further revealed the mechanistic role of ELFN1-AS1 as a competitive endogenous RNA for microRNAs, inducing tumor growth, invasive features, and drug resistance. Additionally, the investigation into the clinical implication of ELFN1-AS1 has demonstrated its potential as a diagnostic, therapeutic, and, notably, prognostic marker. This review provides a comprehensive summary of evidence regarding the involvement of ELFN1-AS1 in cancer initiation and development, highlighting its clinical significance.

## Introduction

Cancer poses a significant threat to human health, and research in this field is rapidly advancing. Exploring the molecular basis of cancer biology has unveiled the intricate molecular network within cells, a valuable guide for investigating potential markers [1, 2]. As the central element of molecular cell networks, the genome is transcribed into ribonucleic acids (RNAs), of which only a small fraction (3%) encodes proteins. Therefore, the majority of RNA transcripts consist of non-coding sequences. However, this does not imply that non-coding RNAs (ncRNAs) lack information or function. Instead, they regulate the cellular physiology and pathogenesis of diseases, particularly cancers, by regulating gene expression at epigenetic, transcriptional, and post-transcriptional levels [3, 4]. Based on their length and shape, ncRNAs can be classified into different classes, including microRNAs (miRNAs), circular RNAs (circRNAs), and long non-coding RNAs (lncRNAs). These three regulatory ncRNAs play crucial roles in various biological processes [5]. miRNAs, consisting of fewer than 200 nucleotides, function both in the nucleus and cytoplasm by regulating gene expression at the post-transcriptional level. This regulation leads to the modulation of malignant cellular features such as proliferation, migration, and invasion [6, 7]. circRNAs derive their name from their distinctive closed-loop shape, which confers robustness against external insults. Their length can vary from 100 to 10,000 nucleotides. circRNAs exert their biological functions through various mechanisms, including competitively sponging miRNAs, transcriptional regulation, and forming regulatory complexes with proteins [5, 8, 9]. Similar to other classes of ncRNAs, many lncRNAs have been identified using gene sequencing technologies. Initially, lncRNAs were a byproduct of gene transcription without regulatory functions [10]. However, further research has elucidated their structure and functions. lncRNA strands are greater than 200 nucleotides and possess a three-dimensional structure that enables them to perform diverse functions, resembling proteins and RNAs. LncRNAs primarily function at the epigenetic, transcriptional, and post-transcriptional levels of gene expression in the cytoplasm and nucleus, regulating transcription, modulating mRNA processes, controlling protein activity, and changing nuclear domains. In addition to these roles, lncRNAs interact with miRNAs, leading to miRNA-induced RNA decay, competition between miRNAs and lncRNAs to regulate specific mRNAs, and the degradation of miRNAs. The latter interaction, miRNA degradation, is the most common. Despite gaining insights into the potential functions of lncRNAs, the biological roles of most lncRNAs still need to be clarified. Therefore, there still exists potential for uncovering novel functions and expanding our understanding of lncRNAs [11–15]. The contribution of different types of lncRNAs to cancers and their potential has been extensively explored in previous studies, paving the way for suggesting novel diagnostic, therapeutic, and prognostic options for cancer management [3, 12, 13, 16, 17]. The extracellular leucine-rich repeat and fibronectin type III domain containing 1-antisense RNA 1 (ELFN1-AS1) is a non-protein coding lncRNA positioned within the intron of the ELFN1 gene, located on chromosome 7p22.3. Experimental evidence has demonstrated that lncRNA ELFN1-AS1 is overexpressed in different tumor samples compared to normal tissues. These findings have revealed that ELFN1-AS1 may play a regulatory role in promoting the malignant characteristics of cancers [18]. Numerous studies have investigated the role of lncRNA ELFN1-AS1 in various cancers, such as colorectal, pancreatic, gastric, lung, ovarian, and ocular cancers [18–24].

This review comprehensively summarizes the studies investigating ELFN1-AS1 and its implicated mechanisms in different cancers. This study elucidates the interplay between ELFN1-AS1 and other molecules, shedding light on their collective effect on tumor malignant characteristics. Additionally, we will discuss the significance of the prognostic models of ELFN1-AS1 in various types of cancers.

## ELFN1-AS1 deregulation in cancer

The compiled studies on the abnormal expression of ELFN1-AS1 in cancers have consistently demonstrated ELFN1-AS1 overexpression in cancer cell lines compared to their normal counterparts. Through laboratory experiments, studies have uncovered the regulatory interplay between ELFN1-AS1 and various cellular molecules, including proteins and RNAs. These interactions have enhanced malignant features in vivo and in vitro (as summarized in Table 1). The following sections will comprehensively overview the biological networks and underlying mechanisms through which ELFN1-AS1 affects cancers.

**Table 1:** Biological functions of ELFN1-AS1 in cancers

Tumor type	Clinical samples	Cell lines	Animals	Reg	In vitro outcomes	In vivo outcomes	Regulatory Axis	Refs.
Gastrointestinal cancers								
CRC	150 pairs	HCT116, HT29, SW480, SW620	BALB/c nude mice	–	↑ Tumorogenesis ↑ Oxaliplatin resistance	↑ Tumor growth	EZH2-DNMT3a/MEIS1	[24]
CRC	–	HCT116, HT29	BALB/c nude mice	Up	Escape NK cell surveillance	↑ Tumor growth	GCN5-SND1/GDF15	[35]
CRC	12 pairs	HCT116, SW480, SW620, HT29, RKO, LoVo	BALB/c nude mice	Up	↑ Proliferation ↓ Apoptosis	↑ Tumor growth	EZH2-FOXP1/TPM1	[27]
CRC	62 pairs	HCT116, SW620, HT29, LoVo	–	Up	↑ Proliferation ↑ Migration ↓ Apoptosis	–	miR-4644/TRIM44	[28]
CC	32 pairs	HCT116, SW480, LoVo, HT29	–	Up	↑ Proliferation ↑ Invasion ↓ Apoptosis	–	miR-191-5p/TRIM14	[29]
CC	45 pairs	SW480, HCT116, SW620	BALB/c nude mice	Up	↑ Proliferation ↑ Migration ↓ Apoptosis	↑ Tumor growth	miR-4270/AURKB	[30]
CC	20 pairs	SW620, HT-29, HCT-116, LoVo, SW480	BALB/c nu/nu mice	Up	↑ Proliferation ↑ Migration ↑ Invasion ↓ Apoptosis	↑ Tumor growth	miR-191-5p/SATB1	[31]
CRC	40 pairs	HT29, HCT116, HCT8, LoVo, SW480, SW620	BALB/c nude mice	Up	↑ Proliferation ↑ Migration ↑ Invasion ↓ Apoptosis	↑ Tumor growth	miR-1250/MTA1	[32]
CRC	40 pairs	SW480, RKO, HCT116	BALB/c nude mice	Up	↑ Proliferation ↑ Migration ↑ Invasion ↓ Apoptosis	↑ Tumor growth	YY1/ELFN1-AS1/TP53/PPP pathway/G6PD	[33]
CC	22 pairs	CaCO-2, SW-480, HCT-116	–	Up	↑ Proliferation ↑ Migration ↑ EMT	–	ERK pathway/EMT	[34]
GC	40 pairs	HGC-27, AGS, MKN74, GTL-16	BALB/c nude mice	Up	↑ Proliferation ↑ Migration ↑ Invasion ↓ Apoptosis ↑ EMT	↑ Tumor growth	miR-211-3p/TRIM29	[21]
GC	–	AGS, NCI-N87, MKN-45, SNU-1	BALB/c nude mice	Up	↑ Proliferation ↑ Migration ↑ Invasion ↓ Apoptosis	↑ Tumor growth	ZBTB16/PI3K/AKT	[37]
PC	–	BxPC-3, SW1990, PANC-1	Nude mice	Up	↑ Proliferation ↑ Migration ↑ Invasion ↑ EMT	↑ Tumor growth	EMT	[23]
EC	31 pairs	EC1, EC109, EC9706, TE1, KYSE70	–	Up	↑ Proliferation ↑ Migration ↑ Invasion	–	miR-183-3p/GFPT1	[41]
Other cancers								
RB	39 pairs	ARPE-19, Y79, SO-RB50, WERI-RB-1	-	Up	↑ Proliferation ↑ Migration ↑ Invasion	-	miR-4270/SBK1	[22]
OC	20 pairs	HO8910, HO8910PM, SKOV3	Nude mice	Up	↑ Proliferation ↑ Migration ↑ Invasion	↑ Tumor growth	miR-497-3p/CLDN4	[19]
NSCLC	117 pairs	-	-	Up	↑ Proliferation ↑ Migration ↑ Invasion	–	miR-497/CCNE1	[20]
OSa	10 pairs	143B, MG63, SW1353	BALB/c nude mice	Up	↑ Proliferation ↑ Migration ↑ Invasion	↑ Tumor growth	miR-138-5p + miR-1291/CREB1/CD206, IL-10	[45]

*CC* colon cancer, *CRC* colorectal cancer, *EC* esophageal cancer, *EMT* epithelial-mesenchymal transition, *GC* gastric cancer, *NSCLC* non-small cell lung cancer, *OC* ovarian cancer, *OSa* osteosarcoma, *PC* pancreatic cancer, *RB* retinoblastoma, *Reg* regulation, *TCGA* the cancer genome atlas, *UM* uveal melanoma, *UP* upregulated

### Colon cancer

Colorectal cancer (CRC) ranks third in incidence and deaths among men and women as of 2022 [25]. Considering the substantial burden CRC imposes on the healthcare system, it is imperative to investigate the underlying mechanisms of CRC development to advance novel methods for disease management [26]. Experiments and analysis conducted by Yimin Li et al. [24] showed that ELFN1-AS1 interacted with DNA methyltransferase 3 alpha (DNMT3a) and enhancer of zeste homolog 2 (EZH2), both of which are involved in histone and DNA methylation processes. These interactions downregulated myeloid ecotype virus insertion site 1 (MEIS1), responsible for DNA damage repair in CRC cells. Consequently, ELFN1-AS1 promoted tumorigenesis in CRC cell lines and enhanced tumor growth in nude mice by regulating the EZH2/DNMT3a/MEIS1 axis, ultimately leading to a poor prognosis. Similarly, Chenyao Li et al. [27] reported that the overexpression of ELFN1-AS1 induced by MYC contributed to tumor growth by enhancing the proliferation and restricting the apoptosis of malignant cells. Overexpressed ELFN1-AS1 suppressed tropomyosin 1 (TPM1) by suppressing EZH2 and the forkhead box P1 (FOXP1) complex.

Ren Lei et al. and Xu Jing et al. [28, 29] conducted similar experiments showing overexpressed ELFN1-AS1 increases tripartite motif-containing 44 (TRIM44) and TRIM14. This upregulation occurs through the competitive binding of ELFN1-AS1 with miR-4644 and miR-191-5p. These interactions promote malignant cell proliferation, migration, and invasion while suppressing apoptosis. Also, Xu Jing et al. indicated that hypoxia, as a feature of colon cancer tumor microenvironment, upregulated the ELFN1-AS1 expression in hypoxic cellular models. Shuangqin Peng et al. Yongjun Du et al. and L-Q Zhai et al. [30–32] concluded that ELFN1-AS1 could upregulate the expression of AURKB, AT-rich sequence binding protein 1 (SATB1), and metastasis-associated protein 1 (MTA1) by sponging miRNAs. As a result, they enhanced proliferation, invasion, and migration and decreased apoptosis in CRC cell lines. In addition, in vivo experiments revealed that the overexpression of ELFN1-AS1 is associated with a larger tumor xenograft in nude mice.In another study, Fahong Wu et al. [33] demonstrated that ELFN1-AS1 activated the pentose phosphate pathway (PPP) by increasing glucose-6-phosphate dehydrogenase (G6PD) expression. Consequently, ELFN1-AS1 could enhance the malignant characteristics of CRC both in vivo and in vitro by interfering with metabolic pathways. Moreover, Liyang Dong et al. [34] revealed that ELFN1-AS1 was aberrantly expressed in colon adenocarcinoma cell lines. Knockdown of ELFN1-AS1 was achieved by treating the cell lines with small interfering RNA against ELFN1-AS1. Downregulated ELFN1-AS1 inhibited the epithelial-mesenchymal transition (EMT) process and extracellular signal-regulated kinase (ERK) signaling pathway. As a result, the suppression of proliferation and migration in vitro was observed. Also, Bin Han et al. [35] showed that ELFN1-AS1 is upregulated in CRC tissues, correlating with poor patient survival. ELFN1-AS1 plays a significant role in CRC by promoting immune escape from natural killer (NK) cell surveillance, by enhancing CRC cells' ability to evade NK cell surveillance both in vitro and in vivo. Knockdown of ELFN1-AS1 decreases colony formation and increases apoptosis in CRC cells when co-cultured with NK cells. Conversely, overexpression of ELFN1-AS1 results in increased colony formation and reduced apoptosis, demonstrating its role in promoting immune escape. Mechanistically, ELFN1-AS1 downregulates the expression of NK cell receptors NKG2D and GZMB. This effect is associated with the activation of the GDF15/JNK pathway. ELFN1-AS1 also enhances the interaction between the proteins GCN5 and SND1, influencing H3K9ac (e.g., histone modifications) enrichment at the GDF15 promoter. The increased GDF15 production in CRC cells further contributes to immune escape by suppressing NK cell cytotoxicity. These findings provide valuable insights into the role of ELFN1-AS1 in CRC. ELFN1-AS1 interacts with key regulatory molecules such as miRNAs and proteins, interferes with DNA repair processes, and crosstalks with cellular pathways such as the ERK signaling pathway and PPP metabolic pathway. These multifaceted processes highlight the diverse functions of ELFN1-AS1 in CRC and provide a foundation for further investigation into its potential as a diagnostic tool and target for therapy.

### Gastric cancer

Despite the declining incidence and mortality rates of gastric cancer (GC), it remains a significant global health concern, with over one million new cases reported annually worldwide. Therefore, there is an urgent need to identify effective therapeutic targets for GC [36]. Jinxi Huang et al. [21] provided evidence suggesting the high expression of ELFN1-AS1 in GC cell lines. Their experiments indicated elevated ELFN1-AS1 upregulates TRIM29 expression by sponging miR-211–3p. This regulatory axis could promote tumor size, facilitate proliferation, migration, and invasion, and suppress apoptosis in experimental assays. Additionally, Shao-Hua Zhuang et al. [37] discovered that ELFN1-AS1 exhibited high expression in GC and was associated with tumor growth and cancer progression. The effect of ELFN1-AS1 was evident through increased proliferation, migration, and invasion and suppressed apoptosis. Functional assays further revealed that ELFN1-AS1 represses zinc finger and BTB domain-containing 16 (ZBTB16), which in turn activates the phosphatidylinositol 3-kinase/protein kinase B (PI3K/AKT) pathway, thereby restoring the invasive nature of GC. In aggregate, elevated expression of ELFN1-AS1 is coupled with its ability to promote GC progression through various molecular pathways, including proteins, miRNAs, and PI3K/AKT signaling pathways, which may hold promise for novel therapeutic targets.

### Pancreatic cancer

Due to the low overall survival rate of pancreatic cancer (PC) patients, it remains one of the leading causes of cancer-related mortality. PC has significantly increased its global burden over the past 25 years [38, 39]. Gang Ma et al. [23] observed that ELFN1-AS1 is overexpressed in PC cell lines compared to normal cells. Gene set variation analysis revealed a close association between ELFN1-AS1 and the EMT process involved in cellular invasion and migration. Furthermore, the knockdown of ELFN1-AS1 exhibited an inhibitory effect on tissue growth in nude mice xenografts. Therefore, further experiments can follow up the therapeutic potential of the ELFN1-AS1 in pancreatic cancer.

### Esophageal cancer

Esophageal cancer (EC) is characterized by a poor prognosis and high mortality rate, highlighting the urgent need for an optimal therapeutic target [40].

Chunyan Zhang et al. [41] found that ELFN1-AS1 is highly enriched in EC cell lines compared to normal tissue. It promotes EC progression by competitively sponging miR-183-3p, thereby increasing the expression of glutamine-fructose-6-phosphate transaminase 1 (GFPT1). This network negatively impacts the OS of EC patients. Thus, ELFN1-AS1 promotes cellular proliferation and invasiveness by regulating the miR-183-3p/GFPT1 axis. The contribution of ELFN1-AS1 to unfavorable prognosis substantiates its potential as a promising target for EC treatment, offering a possibility to improve the outcomes of EC patients.

### Lung cancer

Lung cancer, the second most common neoplasm in males and females, is the deadliest among other cancers. Non-small cell lung cancer (NSCLC) represents approximately 85% of all lung cancers. Therefore, an urgent need for a therapeutic target for NSCLC arises, given its high prevalence among other types of lung cancers [25, 42]. Bin Yang et al. [20] revealed that ELFN1-AS1 has higher expression in NSCLC tissues than in normal paired tissues. The increased expression of ELFN1-AS1 in NSCLC was closely related to advanced TNM stage, lymph node metastasis, and lower OS. Functionally, the knockdown of ELFN1-AS1 significantly impeded the proliferation, migration, and invasion of NSCLC by acting as a sponge for the miR-497/CCNE1 axis. Therefore, the ELFN1-AS1/miR-497/CCNE1 axis may serve as a functional oncogenic pathway in NSCLC, making it a potential target for repressing NSCLC progression.

### Ovarian cancer

Ovarian cancer (OC), a malignant solid tumor originating from ovarian tissues in women, contributes to approximately 12,810 deaths annually and ranks fifth in cancer-related mortality. Hence, understanding the underlying molecular mechanism of OC and developing a reliable biomarker are essential priorities [25]. According to Youkun Jie et al. [19], the expression of ELFN1-AS1 was notably elevated in OC, and subjects with higher levels of ELFN1-AS1 had a poorer prognosis. Depletion of ELFN1-AS1 led to cell proliferation, migration, and invasion and suppressed tumor growth in nude mice, indicating the oncogenic role of ELFN1-AS1 in OC progression. Mechanistically, ELFN1-AS1 downregulates CLDN4 by sponging miR-497-3p. Therefore, targeting the ELFN1-AS1/miR-497-3p/CLDN4 axis is a therapeutic strategy for OC.

### Osteosarcoma

Osteosarcoma (OSa), the most prevalent primary malignant tumor of the bones, primarily develops in long bones such as the humerus, tibia, and femur. OSa has a bimodal age distribution, with a higher incidence in children and adolescents. Despite the application of polychemotherapy and surgical treatment, the survival rate of OSa patients has shown no improvement in recent years [43, 44]. Bangmin Wang et al. [45] proved that the expression of ELFN1-AS1 in OSa cell lines is relatively higher than in normal cells. Tumor-associated macrophages in the tumor microenvironment can have either a protumor (M2) or antitumor (M1 macrophages) phenotype. Functional experiments showed that OSa-derived exosomal ELFN1-AS1 facilitated M2 macrophage polarization by sponging miR-1291 and miR-138-5p. The increased M2 macrophages promote the invasiveness of OSa cells and increase tumor diameter in nude mice xenografts. Moreover, the knockdown of ELFN1-AS1 inhibited the proliferation and invasiveness of OSa cells.

These findings suggest a potential therapeutic axis comprising targets for OSa therapy; however, further explorations are required to develop novel therapeutic methods.

### Retinoblastoma

Retinoblastoma (RB) is the most frequently diagnosed primary ocular malignancy in childhood. If left untreated, it can be lethal. While RB is considered curable in high-income countries, it remains an obstacle in low- and middle-income countries, where more than 80% of RB cases are diagnosed [46–48]. Wanguo Feng et al. [22] investigated the role of ELFN1-AS1 to better understand the underlying mechanisms in RB initiation and progression and identify potential therapeutic targets. The findings revealed that ELFN1-AS1 is upregulated in RB tissues and cell lines, promoting RB cell proliferation and invasiveness by upregulating SBK1 by sponging miR-4270. These findings illustrated that ELFN1-AS1 could be a therapeutic target for RB patients.

## ELFN1-AS1 mechanism of action in cancer

### Interaction with RNAs

**Fig. 1:**
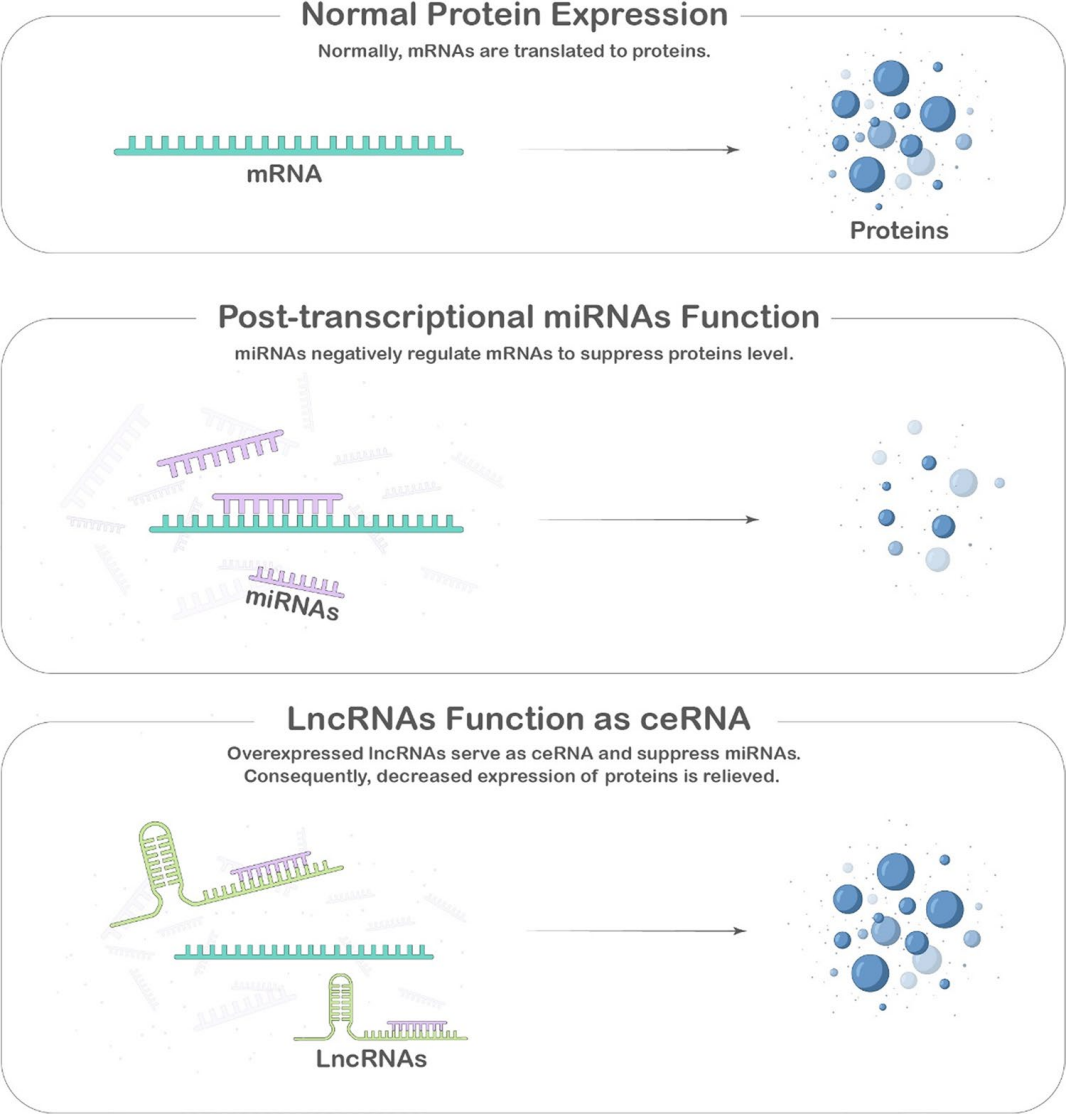
LncRNAs as ceRNAs for miRNAs. Normally, mRNAs are translated into proteins, and miRNAs negatively regulate mRNA levels to suppress protein expression. When lncRNAs are overexpressed, they act as ceRNAs, competing with mRNAs for miRNA binding. This competition relieves the miRNA-mediated suppression on mRNA, leading to increased protein expression. In essence, overexpressed lncRNAs serve as molecular sponges for miRNAs, preventing them from inhibiting their target mRNAs and consequently alleviating the suppression on protein expression

**Fig. 2:**
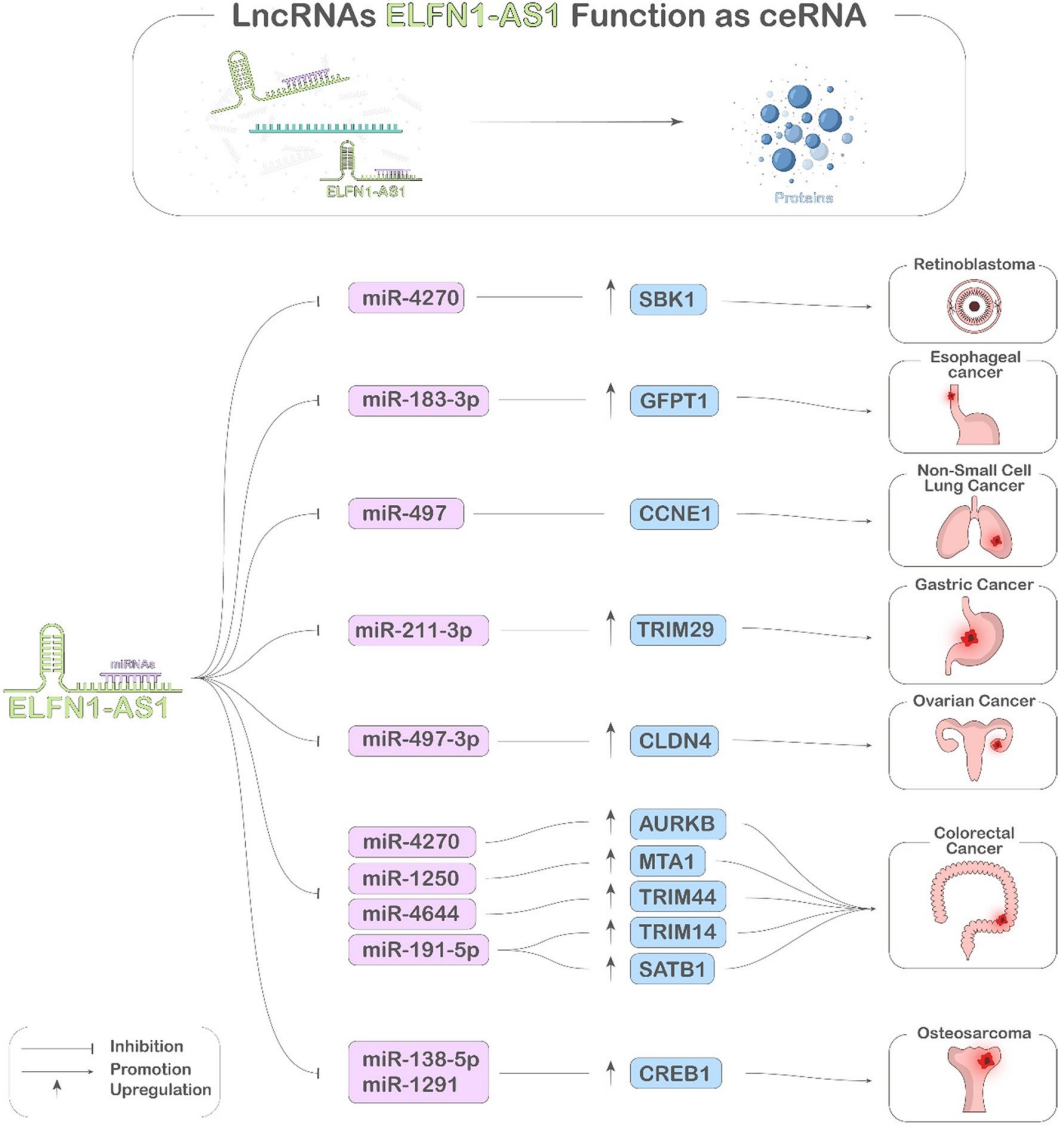
Illustration of the ceRNA Regulatory Axis by ELFN1-AS1. ELFN1-AS1 functions as a ceRNA for miRNAs (depicted in purple boxes), modulating the expression of cellular factors (represented in blue boxes and arrows) and thereby influencing tumor progression

The concept of a competitive endogenous RNA (ceRNA) network was initially hypothesized by Salmena et al. in 2011, which stated that certain RNAs, serving as ceRNAs, can regulate the expression of proteins by competing for shared miRNAs [49–52].LncRNAs, functioning as ceRNAs, can bind to miRNAs and competitively block their activity, attenuating the repressed mRNA and leading to increased translation of mRNA to protein [53] (Fig. 1).This way, lncRNAs can manipulate the progression of various cancers, such as colorectal, ovarian, and hepatocellular cancer, by acting as ceRNAs [54–56].Previous studies found that lncRNA ELFN1-AS1 could negatively regulate miRNAs as a ceRNA mechanism in different cancers and enhance downstream mRNA expression, contributing to the malignant features of tumors. We summarized the regulatory ceRNA axis of lncRNA ELFN1-AS1 in Fig. 2.

### Interaction with proteins and DNA

The studies illustrated that lncRNA ELFN1-AS1 could guide proteins to participate in regulatory networks. Additionally, ELFN1-AS1 could modify DNA methylation. ELFN1-AS1 assists the cellular proteins EZH2 and DNMT3a in locating the promoter of MEIS1 in CRC cells. Enriched ELFN1-AS1-induced EZH2-DNMT3a enhances DNA methylation and mediates trimethylation of Lys-27 in histone 3 (H3K27me3) in the promoter region of MEIS1 to epigenetically repress the transcription of MEIS1. In vivo experiments have shown that decreased levels of MEIS1 suppressed tumor growth in nude mice xenografts [24]. Chenyao Li et al. found that the MYC gene transcriptionally upregulates ELFN1-AS1 in another study. MYC is a dysregulated oncogenic gene whose cancer-causing features are implicated in many malignancies. Mechanistic assays have shown that ELFN1-AS1 recruits EZH2 and forehead box p1 (FOXP1) to alter the promoter region of TPM1. Since TPM1 is a tumor suppressor in CRC, suppression of TPM1 in CRC cells mediates the growth of malignant cells in CRC cell lines and tumor growth in nude mouse xenografts [27, 57]. Shao-Hua Zhuang et al. [37] provided evidence that the enrichment of ELFN1-AS1 induced DNA methylation of the ZBTB16 promoter region by DNA methyltransferases (Dnmts) to repress the expression of ZBTB16. Evidence has shown that ZBTB16 is a tumor suppressor protein. Therefore, the knockdown of ELFN1-AS1 relieves ZBTB16 to inhibit GC cell growth, promote apoptosis in vitro, and suppress tumor xenograft growth in vivo.

### Interaction with cellular pathways

Three studies have identified that ELFN1-AS1 interacts with the ERK and PI3K/AKT signaling pathways and metabolic PPP. The pathways and their interactions with ELFN1-AS1 are briefly explained below.

Extracellular signal-regulated kinase (ERK) is a mitogen-activated protein kinase (MAPK) pathway regulator, which comprises proteins such as RAS, RAF, MEK, and ERK. The MAPK/ERK signaling pathway involves vital cellular processes, including cell proliferation, migration, and invasion. Genetic or epigenetic alterations in proteins of this signaling cascade contribute to the activation of the pathway, which has been found in various types of cancers and renders therapeutic targets [58, 59]. Knockdown of ELFN1-AS1 decreased phosphorylated ERK in cancerous cells, inhibiting proliferation and migration in colon cancer cell lines. This interaction suggests ERK could act as an ELFN1-AS1 downstream target and mediate malignant features. Therefore, considering ELFN1-AS1 can activate ERK and promote malignant features, it should be explored as a potential therapeutic target in future research [34].The PPP is a branch of glycolysis that is involved in the synthesis of NADPH by consuming glucose as the primary substrate. The enzyme G6PD generates NADPH, which is consumed to remove toxic reactive oxygen species in the tumor microenvironments generated during accelerated cellular metabolism or DNA damage of cancerous cells. Therefore, PPP helps cancer cells combat oxidative stress and protect them from death. Evidence has shown that the tumor suppressor p53 (TP53) is a regulator of PPP, as it can inhibit the expression of glucose transporters (GLUT1 and GLUT4). Therefore, TP53 could deprive cancer cells of glucose, the primary substrate for PPP [60–63]. Fahong Wu et al. [33] found that ELFN1-AS1 promotes G6PD activity by inducing the degradation of TP53 protein in CRC. The increased G6PD leads to an accumulation of NADPH. Mechanistic assays demonstrated that the knockdown of ELFN1-AS1 represses CRC cell proliferation, migration, and invasion. Additionally, in vivo experiments indicated that the knockdown of ELFN1-AS1 suppresses xenograft tumors in mice. In summary, ELFN1-AS1 is a crucial PPP upstream regulator affecting CRC progression. The PI3K/AKT signaling pathway is involved in physiological and pathological processes, especially cancers, within the human body. This pathway controls cell survival during cellular stress conditions. Therefore, the role of the PI3K/AKT pathway is significant in tumors since cancer cells inherently proliferate and survive in stressful environments [64]. As explained in the previous section, ELFN1-AS1 suppresses the expression of ZBTB16. Functional assays performed by Shao-Hua Zhuang et al. demonstrated that decreased levels of ELFN1-AS1 activate the PI3K/AKT pathway in GC cell lines. This study does not investigate how ELFN1-AS1 activates the PI3K/AKT pathway. However, we should consider the role of ELFN1-AS1 in activating the PI3K/AKT pathway to promote the tumorigenicity of GC [37].

### Regulation of the EMT process

**Fig. 3:**
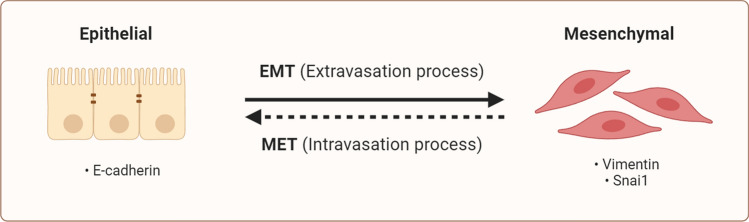
Graphical illuatration of EMT and MET process. E-cadherin and vimentin are the main markers of epithelial and mesenchymal traits of the EMT process

EMT is a conserved cellular process that occurs in different tissue types and developmental processes and participates in carcinogenesis. EMT is involved in cancer initiation and invasion, metastasis to different organs, and therapy resistance. During EMT, epithelial cells are converted to more mesenchymal cell states. During EMT, cells undergo changes in adhesion molecules, enabling them to acquire migratory and invasive properties. The reverse process, mesenchymal-epithelial transition (MET), is associated with the loss of migratory behavior and the adoption of epithelial characteristics. EMT is driven by pleiotropic signaling factors, with parallels between embryonic development, wound healing, fibrosis, and cancer metastasis. EMT transition is orchestrated by specific transcription factors such as Snail, Zeb, Twist, and miRNAs, along with epigenetic and post-translational regulators (Fig. 3). E-cadherin and vimentin are key molecular markers of epithelial and mesenchymal traits, respecticely, during EMT to confirm the process's occurance. Downregulation of E-cadherin is one of the characteristics of EMT, which disrupts adherens junctions. Additionally, overexpressed vimentin, which is involved in the cytoskeleton, contributes to EMT [65–68]. Jinxi Huang et al., Gang Ma et al., and Liyang Dong et al. performed similar experiments on stomach, pancreas, and colon cancers to investigate whether ELFN1-AS1 could alter the EMT process. Mechanistic assays showed that downregulated ELFN1-AS1 in cancerous cells enhanced E-cadherin expression. In contrast, it repressed vimentin expression, which indicates that the EMT process is a downstream effect of ELFN1-AS1 in different gastrointestinal malignancies [21, 23, 34].

## ELFN1-AS1 clinical implication

### Diagnosis

A cancer diagnosis is an essential ring of the cancer management chain since it determines the type of therapy, influencing the patient’s outcome [69, 70]. Therefore, diagnostic biomarkers are of great importance in the cancer management process. Previous sections demonstrated that cancer tissues expressed ELFN1-AS1 aberrantly compared to normal tissues. Therefore, assessing the diagnostic value of ELFN1-AS1 in cancers could be an important hotspot in research. Ji-Xi Liu et al. examined the diagnostic role of ELFN1-AS1 in colon cancer. To determine the diagnostic lncRNAs in colon cancer, tissue samples of patients with colon adenocarcinoma and colonic intraepithelial neoplasia were used to identify five lncRNAs, and the results proved that ELFN1-AS1 was overexpressed. The TCGA database samples validated these findings. The receiver operating curve (ROC) of ELFN1-AS1 had a high area under the curve (AUC) of 0.953 (sensitivity: 100%, specificity: 87.6%), which showed that ELFN1-AS1 could discriminate colon adenocarcinoma from nontumor tissues. Additionally, Yimin li et al. [24] showed that the ROC curve of the combined ELFN1-AS1-MEIS1 had a high AUC of 0.989, which could diagnose CRC patients recruited from the TCGA database.

### Treatment

Approving new therapies targeting molecular networks in cancer, namely, targeted therapy, is a milestone. Targeted therapy refers to targeting specific signaling pathways, proteins, and enzymes in the cell, thereby manipulating oncogenesis [71, 72]. In addition to an optimal therapeutic choice, resistance to current therapies is important, contributing to chemotherapy failure as the main cancer treatment option [73]. Herein, the potential role of ELFN1-AS1 as a therapeutic target in cellular networks for targeted therapy was investigated in the previous sections, and potential targets were proposed. Now, studies investigating the treatment of cancers regarding ELFN1-AS1 as a therapeutic biomarker are discussed.

Previously, Yimin li et al. [24] provided evidence that ELFN1-AS1 interacted with EZH2-DNMT3a to downregulate MEIS1 expression. Western blot assays indicated overexpressed ELFN1-AS1 predisposed CRC cells to oxaliplatin-induced DNA damage. Oxaliplatin is an antitumor chemotherapeutic agent that works by damaging DNA. Therefore, lower levels of MEIS1 induced by ELFN1-AS1 promote CRC cell resistance to oxaliplatin. Yuting Qiu et al. [74] used the Genomics of Drug Sensitivity in Cancer (GDSC) database to estimate the half-maximal inhibitory concentration (IC50) of chemotherapeutic agents in recruited colon cancer patients from the TCGA database. The sensitive response to chemotherapeutic drugs between the low- and high-risk groups (details of the groups are explained in the following section, “Prognosis”) was also compared. Patients in the low-risk score group had more sensitivity to common therapeutic agents. Cervical cancer (CCa) is the second most common cancer in women globally, with over 570,000 new cases and 310,000 deaths each year. About 85% of cases occur in developing countries. Despite the diagnostic and screening advancements, the CCa-related morbidity rate, mortality rates, and poor prognosis persist [75]. Regarding the therapeutic approaches, Jinhui Liu et al. [76] developed a risk score based on ELFN1-AS1 to determine the sensitivity to chemotherapeutic agents in CCa patients. The results showed that patients in the high-risk score group had a more sensitive response to three common chemotherapeutic agents (methotrexate, vinorelbine, and paclitaxel).

These findings highlight the role of ELFN1-AS1 in resistance to chemotherapeutic agents, and it should be considered a potential biomarker to improve cancer therapy.

### Prognosis

**Fig. 4:**
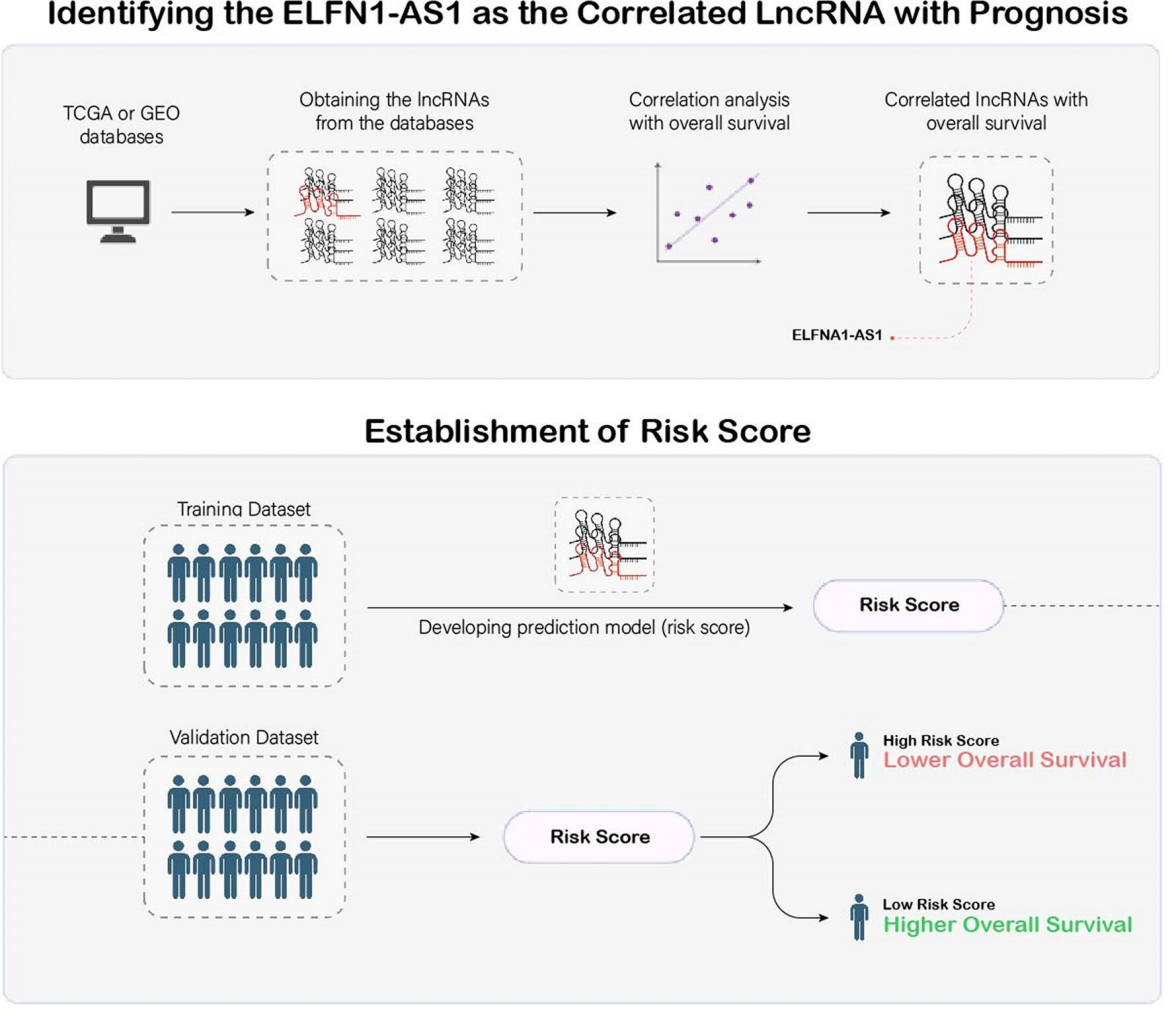
Workflow of Risk Score Signature Establishment. The study employed statistical methods, including correlation analysis, to explore the association between lncRNAs and patients' overall survival, with a focus on ELFN1-AS1. Correlated lncRNAs, including ELFN1-AS1, underwent multivariate regression analysis to determine the lncRNA coefficient. The risk score signature was established by computing a linear combination of lncRNA levels multiplied by their respective coefficients. The datasets or clinical samples were divided into training and validation sets. The risk score signature's performance was initially established in the training set and subsequently validated in an independent set. Each patient received a unique score, and based on the median score, the patients were stratified into high- or low-risk groups

Regarding the advancement in cancer therapy, the main question is how targeted therapy improves a patient’s OS. We explored the underlying mechanisms in the “ELFN1-AS1 Deregulation in Cancers” section above. Most studies indicated that higher expression of ELFN1-AS1 was associated with poor clinical prognosis, as summarized in Table 2. However, some studies utilized different methods to examine the prognostic value of ELFN1-AS1 in cancers. In the following section, we will describe the methodology of those studies and corresponding values in the prognosis prediction (Table 2). The availability of public cancer-related datasets such as Gene Expression Omnibus (GEO) and The Cancer Genome Atlas (TCGA) has paved the way for many investigations into gene expression and other relevant clinical features in a wide range of cancers. Studies used statistical methods like correlation analysis to find the association between lncRNAs and patients’ prognoses. Then, correlated lncRNAs (consisting of ELFN1-AS1) were entered in multivariate regression analysis to calculate the lncRNA coefficient. A linear combination of lncRNA levels multiplied by the corresponding coefficient established the risk score signature. Data from datasets or clinical samples are divided into training and validation sets by a specific ratio. First, the risk score signature was established in the training set, and the performance of the signature was tested in the validation set. Accordingly, each patient was assigned a distinct score, and based on the median score of the entire group, patients were divided into high- or low-risk groups. Moreover, some studies investigated potential independent prognostic factors other than the risk score and combined them with the risk score to establish a clinical nomogram. The workflow of ELFN1-AS1-based risk score establishment is illustrated in Fig. 4. Three studies [74, 77, 78] explored ferroptosis-related lncRNAs in CRC patients from the TCGA dataset. Ferroptosis is an iron-dependent programmed cell death that is different from other types of cell death, such as apoptosis or necrosis [79]. They incorporated the risk score with clinicopathological variables, of which age, sex, and tumor staging were the most relevant variables, and a clinical nomogram was developed. The nomogram predicted the patients' OS after one, three, and 5 years. The nomogram for a five-year OS outperformed the other models, and the AUC of a five-year nomogram for all of the studies was approximately 0.8 in validation cohorts. Patients with high-risk scores had a lower OS.

**Table 2:** Characteristics of ELFN1-AS1 prognostic models

Tumor type	Data source and sample size	lncRNA type	Signature	Nomogram	Prognostic and diagnostic value (AUC)	Refs.
CC	TCGA: 39 normal and 379 CC cases FerrDb: 259 genes TIMER and CIBERSORT: 22 immune cells	Ferroptosis-related	Prognostic risk score: 15 lncRNAs	Age, sex, stage, TNM, risk score	1-, 3-, and 5-year OS in training set: 0.796, 0.828, and 0.866 1-, 3-, and 5-year OS in testing set: 0.668, 0.724, and 0.856	[77]
CRC	TCGA: 51 normal and 582 CRC cases FerrDb:382 genes Clinical sample: 30 pairs	Ferroptosis-related	Prognostic risk score: 4 lncRNAs	Age, T, stage, risk score	1-, 3-, and 5-year OS: 0.736, 0.710, and 0.746	[78]
CC	TCGA: 355 COAD cases ICGC:134 COAD cases GDSC: Drug sensitivity	Ferroptosis-related	Prognostic risk score: 9 lncRNAs	Age, stage, risk score	1-, 3-, and 5-year OS in original set: 0.701, 0.785, 0.821 1-, 3-, and 5-year OS in testing set: 0.701, 0.751, 0.833 Nomogram C-index: 0.801	[74]
CRC	TCGA: 51 normal and 644 CRC cases ImmLnc: 2617 genes Clinical sample:10 pairs	Immune-related	Prognostic risk score: 7 lncRNAs	–	OS in entire set: 0.855	[80]
OSa	TCGA: 85 cases Molecular Signature: 331 genes	Immune-related	Prognostic risk score: 6 lncRNAs	–	5-year OS in train set: 0.937 5-year OS in test set: 0.797 5-year OS in entire set: 0.879	[81]
CCa	TCGA: 257 cases Molecular Signatures: 331 genes GDSC: Drug sensitivity	Immune-related	Prognostic risk score: 6 lncRNAs	age, stage, grade, histological type, risk score	1-, 3-, and 5-year OS in training set: 0.775, 0.748, 0.746 1-, 3-, and 5-year OS in testing set: 0.785, 0.689, 0.681 1-, 3-, and 5-year OS in entire set: 0.812, 0.635, 0.603	[76]
DLBCL	GEO: 709 cases TMUCIH: 160 cases GeneCards: 2025 genes	Epigenetic-related	Prognostic risk score: 9 lncRNAs	Age, LDH, ECOG, GCB vs. non-GCB, extranodal sites, risk score	1-, 3-, and 5-year OS in training set: 0.765, 0.780, 0.760 1-, 3-, and 5-year OS in testing set1: 0.597, 0.598, .615 1-, 3-, and 5-year OS in training set2: 0.686, 0.753, 0.760	[82]
CC	TCGA: 452 patients GEO: 177 patients	N7-Methylguanosine-related	Prognostic risk score: 8 lncRNAs	Age, TNM, risk score	1-, 3-, and 5-year OS in training set: 0.671, 0717, 0.692 1-, 3-, and 5-year OS in testing set: 0.679, 0.617, 0.648	[83]
ECa	TCGA: 208 normal and 35 ECa cases 96 genes DrugBank: Drug data	DNA methylation-related	Prognostic risk score: 2 lncRNAs	–	1-, 3-, and 5-year OS in training set: 0.690, 0.590, 0.630 1-, 3-, and 5-year OS in testing set: 0.690, 0.590, 0.630 1-, 3-, and 5-year OS in entire set: 0.730, 0.660, 0.67	[84]
CRC	TCGA: 387 CRC cases GEO: 546 cases GeneCards: 133 genes	Pyroptosis-related	Prognostic risk score: 4 lncRNAs	–	1-, 3-, and 5-year OS in training set: 0.700, 0.698, 0.674 1-, 3-, and 5-year OS in testing set: 0.648, 0.608, 0.601	[85]
HCC	TCGA: 374 HCC and 50 normal cases HADb: 232 genes	Autophagy-related	Prognostic risk score: 7 lncRNAs	TNM, risk score	1-, and 3-year OS in entire set: 0.785, 0.710	[87]

*AUC* area under curve, *CC* colon cancer, *CCa* cervical cancer, *CIN* colonic intraepithelial neoplasia, *COAD* colon adenocarcinoma, *CRC* colorectal cancer, *DLBCL* diffuse large B-cell lymphoma, *ECa* endometrial cancer, *ECOF* Eastern Cooperative Oncology Group, *GCB* germinal center B-cell-like, *GDSC* Genomics of Drug Sensitivity in Cancer, *HADb* human autophagy database, *HCC* hepatocellular carcinoma, *LDH* lactate dehydrogenase, *OS* overall survival, *OSa* osteosarcoma, *TCGA* The Cancer Genome Atlas

Three studies [76, 80, 81] explored immune-related lncRNAs in CCa, CRC, and OSa patients from the TCGA dataset. The established risk score derived from the training set had moderate to high accuracy in predicting OS. Only one study [76] established a clinical nomogram in the CCa dataset incorporating risk score and other independent prognostic predictors (including histological type, stage, grade, and age). In contrast with ferroptosis-related nomograms in CRC, immune-related nomograms performed better in predicting one-year OS. Xiaoxuan Wang et al. [82] constructed a 9-lncRNA (consisting of ELFN1-AS1) epigenetic-related risk signature derived from GEO (GSE10846 dataset) and validated it in both the TMUCIH cohort and GEO (GSE31312 dataset). The risk signature better predicted the five-year OS of diffuse large B-cell lymphoma (DLBCL) patients. Age, lactate dehydrogenase (LDH) level, cell-of-origin, extranodal sites, and Eastern Cooperative Oncology Group performance status were correlated clinicopathological variables used in nomogram construction. Sheng Yang et al. [83] identified eight N7-methylguanosine-related lncRNAs (consisting of ELFN1-AS1) and established a risk signature derived from TCGA and validated it in GEO (GSE17536 dataset) database patients with colon cancer. Based on the risk score and cluster analysis, patients were divided into three groups, of which group 1 had the worst prognosis. The AUCs of the risk signatures are provided in Table 2. A clinical nomogram was also created by integrating risk score, age, and TNM stage. Lu Cao et al. [84] selected two DNA methylation-related lncRNAs (ELFN1-AS1 and ZNF132) in endometrial cancer (ECa). The TCGA database established and validated the DNA methylation-related lncRNA prognostic risk signature. Patients were dichotomized into low- and high-risk groups, of which the high-risk group had a worse prognosis than the low-risk group. The risk signature performed better in the prediction of one-year OS. Sijun Chen et al. [85] developed a prognostic signature of four pyroptosis-related lncRNAs in CRC. Pyroptosis is a form of inflammatory cell death triggered by inflammasomes [86]. The risk signature was derived from the TCGA database and validated by patients from the GEO (GSE39582 dataset) database. The prognostic signature could subdivide patients into low- and high-risk groups, of which the latter had worse OS than the low-risk group. The signature better predicted one-year OS in both the training and validation sets. Yu Jia et al. [87] created a prognostic signature of four autophagy-related lncRNAs (consisting of ELFN1-AS1) in HCC, which is the third cause of cancer-related deaths [15]. Autophagy is a highly conserved recycling process that maintains cellular homeostasis [88]. The risk signature divided patients into low- and high-risk groups based on the median risk score. The high-risk group had a worse OS. Additionally, a nomogram incorporating risk score and TNM staging was constructed. The signature predicted the one-year OS better than the three-year OS.

ELFN1-AS1-related predictive risk signatures could predict patient OS in different cancers, which reminds us that we can rely on ELFN1-AS1 significance as a promising prognostic biomarker in future investigations.

## Summary and concluding remarks

Research advancement has led to an increasing number of investigations on lncRNAs in the oncology field of research, and evidence on the underlying mechanism of action of lncRNAs contributing to cancer progression is increasing. lncRNAs do not code proteins, but their ability to regulate downstream effectors in cellular networks highlights their promising role. Multiple in vivo and in vitro experiments have proven that lncRNAs can manipulate the malignant features of various cancers. Therefore, considering ELFN1-AS1 as a cancer diagnosis, treatment, and outcome biomarker seems compelling.

LncRNA ELFN1-AS1 is a protumor lncRNA, and many studies are dedicated to unraveling the various roles of ELFN1-AS1 in cancer biology. ELFN1-AS1 has been investigated in different cancers, particularly in gastrointestinal (GI) tract malignancies such as colorectal, gastric, esophageal, liver, and pancreatic cancer. Nonetheless, its involvement beyond the GI tract has been studied in lung, ovarian, endometrial, skeletal, hematological, and ocular malignancies. Studies have confirmed the oncogenic contribution of ELFN1-AS1 through different mechanisms, such as interactions with pathways, proteins, DNAs, and particularly RNAs. Interaction with RNAs via ceRNA is the most frequently repeated mechanism in papers and highlights that ELFN1-AS1 may have a promising role in cancer therapy.

**Fig. 5:**
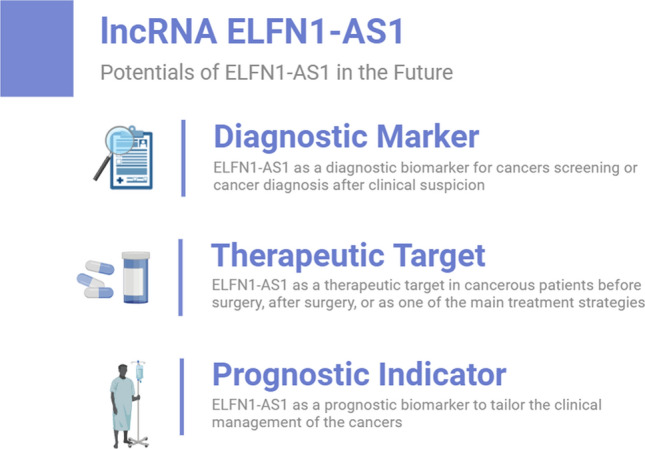
Future prospective of ELFN1-AS1

Regarding clinical implications, some studies have investigated the diagnostic role of ELFN1-AS1 and showed that ELFN1-AS1 may help clinicians diagnose cancers. Moreover, drug sensitivity analysis also indicated that the tumoral tissue response to chemotherapeutic agents could be adjusted by ELFN1-AS1 and demonstrated that ELFN1-AS1 interferes with the response to therapy in cancers. However, investigations have predominantly focused on the prognostic role of ELFN1-AS1 in cancers. Different ELFN1-AS1-based risk models were established, and the results showed that the models had moderate to high accuracy in predicting patient survival after one, three, and 5 years. Additionally, studies have explored the diagnostic, therapeutic, and prognostic role of ELFN1-AS1 and highlighted its importance. However, ELFN1-AS1 needs more clinical exploration for implementation in clinical practice. Potential roles of ELFN1-AS1 are depicted in Fig. 5.

## Data Availability

The original contributions presented in the study are included in the article. Further inquiries can be directed to the corresponding author.
